# A Novel Processing-Free Method for RNAseq Analysis of Spontaneous Sputum in Chronic Obstructive Pulmonary Disease

**DOI:** 10.3389/fphar.2021.704969

**Published:** 2021-08-19

**Authors:** Francesca Ricci, Michele Bassi, Cathy M. McGeough, Gera L. Jellema, Mirco Govoni

**Affiliations:** ^1^ Global Clinical Development, Personalised Medicine and Biomarkers, Chiesi, Parma, Italy; ^2^ Almac Diagnostic Services, Craigavon, Northern Ireland, United Kingdom

**Keywords:** chronic obstructive pulmonary disease, spontaneous sputum, inflammation, RNAseq, non-invasive

## Abstract

**Background:** Assessments of airways inflammation in patients with chronic obstructive pulmonary disease (COPD) require semi-invasive procedures and specialized sample processing know-how. In this study we aimed to set up and validate a novel non-invasive processing-free method for RNA sequencing (RNAseq) of spontaneous sputum samples collected from COPD patients.

**Methods:** Spontaneous sputum samples were collected and stabilized, with or without selection of plugs and with or without the use of a stabilizer specifically formulated for downstream diagnostic testing (PrimeStore® Molecular Transport Medium). After 8 days storage at ambient temperature RNA was isolated according to an optimized RNAzol® method. An average percentage of fragments longer than 200 nucleotides (DV_200_) >30% and an individual yield >50 ng were required for progression of samples to sequencing. Finally, to assess if the transcriptome generated would reflect a true endotype of COPD inflammation, the outcome of single-sample gene-set enrichment analysis (ssGSEA) was validated using an independent set of processed induced sputum samples. Results: RNA extracted from spontaneous sputum using a stabilizer showed an average DV_200_ higher than 30%. 70% of the samples had a yield >50 ng and were submitted to downstream analysis. There was a straightforward correlation in terms of gene expression between samples handled with or without separation of plugs. This was also confirmed by principal component analysis and ssGSEA. The top ten enriched pathways resulting from spontaneous sputum ssGSEA were associated to features of COPD, namely, inflammation, immune responses and oxidative stress; up to 70% of these were in common within the top ten enriched pathways resulting from induced sputum ssGSEA.

**Conclusion:** This analysis confirmed that the typical COPD endotype was represented within spontaneous sputum and supported the current method as a non-invasive processing-free procedure to assess the level of sputum cell inflammation in COPD patients by RNAseq analysis.

## Introduction

Sputum induction and its subsequent processing has become the gold standard clinical tool for accessing airway inflammation in chronic obstructive pulmonary disease (COPD) (Lacy et al., 2005; Dragonieri et al., 2009). In the last 20 years induced sputum (IS) sampling procedure has been extensively standardized (Paggiaro et al., 2002). The technique is generally well-tolerated and safe (Bathoorn et al., 2007; Tangedal et al., 2014) but remains a semi-invasive procedure (Koutsokera et al., 2013) that may create discomfort in COPD patients (Taube et al., 2001; Makris et al., 2006). In addition, its use is limited to specialized centers because it is technically demanding, time consuming and requires trained staff (Paggiaro et al., 2002). Considering that patients with COPD and in particular those with a chronic bronchitis phenotype can easily produce spontaneous sputum (SS), this may represent a suitable alternative to IS. SS is reported as a totally safe and non-invasive technique for the identification of pulmonary biomarkers (Koutsokera et al., 2013). However, cell viability and levels of selected inflammatory markers in SS and IS samples have been compared, obtaining controversial results (Bhowmik et al., 1998; Tangedal et al., 2014). Whether SS and IS samples could be used interchangeably for accessing inflammatory mediators in the lung remains unclear and further investigation has been recommended (Paggiaro et al., 2002; Tangedal et al., 2014).

High throughput transcriptomics techniques have been shown to be particularly useful to assess the level of inflammation in COPD (Berge et al., 2014; Betts et al., 2014; Sridhar et al., 2019). This approach can provide a global picture of complex inflammatory conditions which would be otherwise missed when measuring target biomarkers at protein level. Sputum is a challenging matrix for RNA extraction and generally leads to RNA of low quantity and quality with respect to RNA isolated from other biological samples (Vanspauwen et al., 2014; Paska et al., 2017; Paska et al., 2019). Some studies have investigated the inflammatory conditions in COPD by gene expression analysis of IS samples (Watz et al., 2013; Baines et al., 2018; Govoni et al., 2020). In order to optimize the isolation protocol and improve yield and purity of IS RNA, different collection and processing methods have been evaluated (Vanspauwen et al., 2014; Paska et al., 2017; Paska et al., 2019; Frøssing et al., 2020).

In contrast to IS, no studies to our best knowledge have investigated the suitability of SS samples in COPD to derive appropriate RNA for gene expression analysis. In the present study we developed a processing-free method to stabilize RNA at ambient temperature after SS collection from COPD donors. This was followed by a wet lab optimization protocol aimed to isolate RNA with sufficient quantity and quality for downstream sequencing analysis. To assess whether this new methodology accurately reflects the level of sputum cell inflammation, the outcome of single sample gene set enrichment analysis (ssGSEA) obtained from SS analysis was validated by using an independent set of processed IS samples (Govoni et al., 2020) from COPD patients.

## Materials and Methods

### Overall Study Design

**FIGURE 1:**
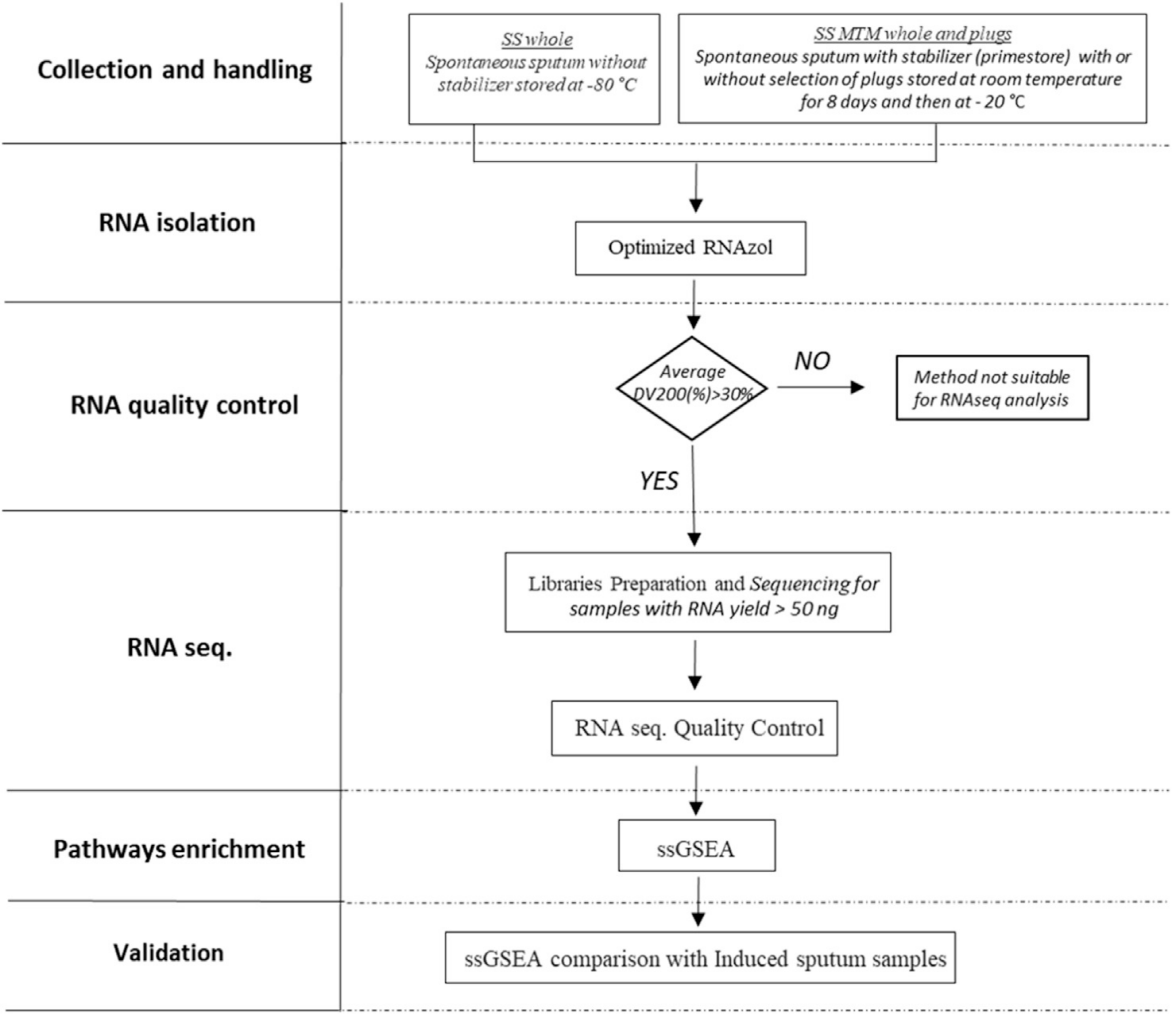
Spontaneous sputum samples without stabilizer (*SS_whole*) and SS with stabilizer (*SS_MTM*) were collected with (*SS_MTM_plug*) or without (*SS_MTM_whole*) selection of plugs. RNA was extracted using an optimized RNAzol® protocol. An average DV_200_ above 30% was considered suitable for progression to RNAseq analysis. Only samples with RNA content higher than 50 ng were submitted to library preparation and sequencing. Finally, the outcome of ssGSEA was validated with ssGSEA results from an independent set of processed induced sputum.

The overall study design is shown in Figure 1. Different processing-free collection and handling methods (with or without stabilizer, and with or without selection of plugs) were tested to identify a suitable method for RNAseq analysis of spontaneous sputum. An adapted and optimized RNAzol® protocol was used for recovery of total RNA. Collection and handling methods generating RNA with an average percentage of fragments longer than 200 nucleotides [Distribution Value (DV_200_)] above 30% were considered suitable for progression to RNAseq analysis. If sufficient quality criteria were not met the method would be reported as not suitable for RNAseq analysis. Only samples from methods passing the prespecified quality metrics and with RNA content higher than 50 ng were submitted to library preparation and sequencing. Finally, to assess if the transcriptome generated from a specific collection and handling method would reflect a true endotype of COPD inflammation, the outcome of ssGSEA was validated against the transcriptome of an independent set of processed induced sputum samples from COPD patients.

### Sputum Collection and Handling

*SS without stabilizer (SS_whole);* samples were collected from a multicenter study, the results for which have been previously reported (Singh et al., 2019; Govoni et al., 2020). Briefly, at screening visit, patients were instructed to blow their noses and rinse their mouths out with water before expectorating sputum into a sterile pot. After collection samples were immediately stored at −80°C at the clinical site until shipment to the analytical laboratory (Almac Diagnostics, Craigavon, United Kingdom) in dry ice conditions where they were then stored at –80°C until analysis.

*SS with stabilizer (SS_MTM);* samples were collected using PrimeStore® Molecular Transport Medium (MTM; Longhorn Vaccines and Diagnostics, United States), a clinical sample collection system specifically formulated for downstream molecular diagnostic testing. Patients were instructed to blow their noses and rinse their mouth out with water before expectorating sputum into a sterile pot (15 ml Nalgene; VWR). Two handling methods have been used. The first, 500 μl of sputum plugs were selected and transferred in a PrimeStore® tube (Longhorn Vaccines and Diagnostics) containing 1.5 ml of MTM (*SS_MTM_plug*). The second, 1.5 ml of MTM (the content of a PrimeStore® tube) was poured into the sterile pot containing the spontaneous sputum collected without prior selection of plugs (*SS_MTM_whole*). Samples were shipped from the clinical site to the analytical laboratory (Almac Diagnostic Services, Craigavon, United Kingdom) at ambient temperature, stored in ambient conditions for 8 days post collection and then placed at –20°C prior to analysis.

### RNA Isolation

RNAzol isolation method allows for extraction of RNA without the use of a chloroform phase separation step. The RNAzol protocol for recovery of total RNA was adapted and optimized as follows: a volume of 400 µL of sputum collected from *SS_whole, SS_MTM_plug* or *SS_MTM_whole* was mixed with 1 ml of RNAzol and incubated for 15 min at room temperature. Following centrifugation at 16,000 × g for 15 min, the DNA, proteins and most polysaccharides were pelleted at the bottom of the tube. The supernatant was recovered, and 1 ml volume of isopropanol was added to precipitate the RNA. Following a series of ethanol wash steps, the crude RNA was quantified using nanodrop™ (Thermo Fisher Scientific, Massachusetts, United States) and purified through incubation with DNaseI followed by filter purification using the RNeasy® MinElute® spin column (Qiagen, Hilden, DE) to yield total RNA in a volume of 14 µL (molecular grade water). Samples were extracted in triplicate to maximise the RNA yield. The extracted RNA samples were then assessed for concentration and purity using the Nanodrop 1000 spectrophotometer (Thermo Fisher Scientific, Massachusetts, United States). Quality was assessed using the Agilent Bioanalyser 2100 system (Agilent, Santa Clara, United States). Fluorescent dye-based quantification of RNA (Qubit, Life Technologies) was utilized to confirm the presence of RNA.

### Library Preparation and Sequencing

Only samples from collection and handling methods passing the prespecified quality metrics (average DV_200_ > 30%) and with individual RNA content higher than 50 ng were submitted to library preparation and sequencing.

Libraries were prepared using KAPA RNA HyperPrep Kit with RiboErase (HMR), (Roche, Basilea, CH) using the manufacturers protocol with an input of 50–100 ng RNA. All libraries were quality assessed for fragment size (Bioanalyser) and quantified using the NEBNext Library Quant kit (New England Biosciences, Herts, United Kingdom). Normalized libraries were loaded onto a NovaSeq SP flow cell (Illumina, SanDiego, US) and sequenced to a total read length of 2 × 76 bp. The quality of the sequencing was confirmed for number of clusters passing filter, Q30 scores, error rate, cluster density and read distribution. Raw sequence data in FASTQ format were input to QC assessment steps hosted on the DNAnexus cloud platform. Basic sequencing metrics such as GC content were calculated from unaligned reads using FastQC (Leggett et al., 2013). Read alignment to the human reference genome GRCh38 was then performed using StarAlign (Dobin et al., 2013), with outputs used to calculate post-alignment QC metrics such as duplication rate [calculated using Picard MarkDuplicates (http://broadinstitute.github.io/picard/)] and coverage of the housekeeping genes.

### Alignment and Expression Calculation

Read alignment was performed using StarAlign (Dobin et al., 2013) to the human reference genome GRCh38. Only uniquely mapped reads were output for downstream analysis and all other reads discarded. Sequences with >=3 base mismatches (against reference) were also discarded. In addition, gene counts were output for downstream analysis. StringTie (Pertea et al., 2015) was used to generate gene expression data, represented as Fragments Per Kilobase per Million fragments mapped (FPKM), Transcripts per million (TPM), and per gene coverage for each gene present in the human (GRCh38) ENSEMBL annotation file. The flag “–B” specified to ensure results are output in a single tab separated file, and the option “--rf” was specified to ensure reads were treated as an fr-firststrand stranded library. To generate the gene level expression, all exons that can be a part of any of the transcripts for that gene were summed.

### ssGSEA Pathways Enrichment Analysis

Functional enrichment analysis was performed using ssGSEA, a projection methodology described in Barbie et al., 2009 (Barbie et al., 2009) to project each sample within a data set onto a space of gene set enrichment scores. Gene sets used for the analysis were part of MSigDB (Subramanian et al., 2005): GO Biological Processes (C5), Hallmarks gene sets (H) and Biocarta pathways (subset of curated gene sets (C2) (Liberzon et al., 2015).

The outcome of ssGSEA was compared between *SS_MTM_plug* and SS_*MTM_whole* samples and between *SS_MTM* and an independent set of processed induced sputum samples collected in a previous study from COPD patients (Govoni et al., 2020).

### Statistical Analysis

Correlation analysis and reduction of dimensionality of the datasets by PCA was performed to evaluate differences between SS_*MTM_plug* and SS_*MTM_whole* expression results.

Pearson method was used to evaluate the correlation between expression results. Analyses were performed in R version 4.0.2 (R Core Team, 2020).

## Results

### Study Samples

Samples were obtained from male or female subjects with a diagnosis of COPD who were able to generate spontaneous sputum and were under treatment with inhaled single or dual long-acting bronchodilators [β2 agonist (LABA) and/or muscarinic antagonist (LAMA)] with or without inhaled corticosteroids (ICS). *SS_whole* and induced sputum validation samples were obtained from 10 and 46 patients, respectively, enrolled in a multi-center trial whose population was previously reported (Singh et al., 2019; Govoni et al., 2020); Caucasian; mean age 66; 70% males with mean (SD) time since first COPD diagnosis of 9.3 (4.7) years and post-bronchodilator predicted FEV1 of 50 (12) %. *SS_MTM_plug* and *SS_MTM_whole* paired samples were obtained from 10 patients of similar clinical characteristic to those providing *SS_whole* and induced sputum validation samples; Caucasian; mean age 62; 70% males with mean (SD) time since first COPD diagnosis of 18.2 (13.1) years and post-bronchodilator predicted FEV1 of 54 (20) %. All patients provided written informed consent prior to any study-related procedure.

### RNA Isolation

Following RNAzol® extraction only spontaneous sputum samples stabilized in MTM generated RNA with an average DV_200_ > 30%. Thus, stabilized samples from both collection and handling methods (with or without selection of plugs) were deemed suitable for progression to library preparation and sequencing. Specifically, the average DV_200_ ± S.E.M. for *SS_MTM_plug* and *SS_MTM_whole* samples was 42.0% ± 4.8 and 36.4% ± 6.3, respectively, whereas the DV_200_ ± S.E.M. of *SS_whole* samples was 27.9% ± 6.5. Therefore, samples from the collection method that does not consider the use of any stabilizer were not considered appropriate for progression to RNAseq analysis (Supplementary Table S1).

### Next Generation Sequencing (NGS)

RNA extracted by seven out of ten *SS_MTM_plug* and *SS_MTM_whole* sample pairs were of sufficient quantity (>50 ng) to proceed to downstream NGS testing (Supplementary Table S1). The DV_200_ average of the seven sample pairs remained higher than 30%. All samples generated libraries which met the required quality control metrics (average concentration±SD 51 ± 44nM; fragment size±SD 427 ± 34bp) and were suitable for sequencing. Sequencing run QC metrics were reached and an average of 100 million reads were achieved. Sequencing data from both the RNA derived from the *SS_MTM_plug* and *SS_MTM_whole* met the quality requirements for bioinformatics RNAseq analysis (Supplementary Table S2). Only one sample pair returned lower mapping rates and housekeeping gene coverage than expected, indicative of a poorer quality sample in both collection and handling methods (DV_200_ = 5 and 19% for *SS_MTM_plug* and *SS_MTM_whole*, respectively). The dataset can be found in the online repository: GEO Series accession number (GSE175829).

### Comparison of Gene Expression Profiles Observed in *SS_MTM_plug* and *SS_MTM_whole*: PCA and Correlation Analysis

**FIGURE 2:**
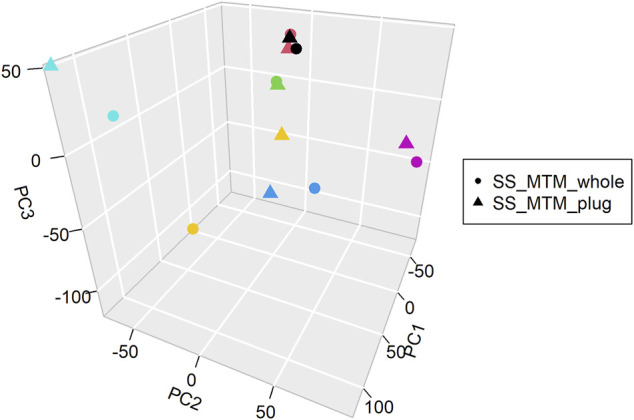
Principal component analysis (PCA) of the RNA-seq dataset for *SS_MTM_plugs* and *SS_MTM_whole* samples. Each color represents a paired sample (plug and whole).

PCA analysis of the overall gene expression profiles (Supplementary Table S3) showed that there was no major distinction between the gene expression distributions of samples collected with or without separation of plugs (*SS_MTM_whole* and *SS_MTM_plug*) (Figure 2). Sample pairs grouped together, except for the pair that was found to have lower mapping rates in NGS analysis and poorer quality (Figure 2, yellow pair). Correlation results showed that there was a significant high correlation (*r* = 0.878–0.989 with a *p*-value close to zero) between the transcriptomic profile of all the sample pairs. Overall, these results suggested comparability of the whole expression profile between the *SS_MTM_whole* and *SS_MTM_plug* samples. This supports the use of either the collection and handling methods (with or without selection of plugs) for deriving RNA gene expression data by NGS.

### Single Sample Gene Set Enrichment Analysis (ssGSEA)

ssGSEA analysis was performed to confirm that the RNASeq data from SS samples stabilized in MTM with or without plugs selection were representative of an expected inflammatory expression profile for COPD patients. ssGSEA calculates a separate enrichment score for each pairing of sample and gene set, independent of phenotype labelling. In this manner, ssGSEA transforms a single sample’s gene expression profile into a gene set enrichment profile. As a gene set’s enrichment score represents the activity level of the biological process in which the gene set’s members are coordinately up- or down-regulated, the results that are of interest have high enrichment scores. Therefore, a median of the enrichment scores was calculated for all samples and subsequently sorted from high to low. ssGSEA results (Supplementary Table S4 in Supplementary Material) showed an excellent match between *SS_MTM_whole* and *SS_MTM_plug* samples with more than 80% of the top 10 pathways identified using different gene sets (GO biological processes, Hallmarks gene sets and Biocarta pathways) in common. For validation purposes, ssGSEA was also applied to an independent data set generated by Affymetrix® GeneChip® HG-U133 Plus 2.0 microarray from IS samples at baseline of a cohort of COPD patients with similar clinical characteristics (Govoni et al., 2020) (GEO Series accession number GSE133513). Comparison of ssGSEA between SS stabilized in MTM and IS samples showed that within different gene sets (GO biological processes, Hallmarks gene sets and Biocarta pathways) key pathways associated to features of COPD inflammation (e.g. interleukins and interferon response) had top enrichment scores for both SS and IS samples. In addition, the top 10 enriched pathways showed a straightforward overlap between SS and IS with up to 70% of pathways in common, for GO biological processes, Hallmarks and Biocarta pathways, respectively; all belonging to key COPD pathways (Table 1).

**TABLE 1:** Common GO biological processes, Hallmarks and Biocarta pathways within the top ten enriched pathways for spontaneous sputum and induced sputum.

GO Biological Processes
T cell activation via T cell receptor contact with antigen bound to MHC molecule on antigen presenting cell
Cotranslation protein targeting to membrane
Establishment of protein localization to endoplasmatic reticulum
Positive regulation of interleukine 6 biosynthetic process
**Hallmarks gene sets**
TNFα signaling via NFkB
Reactive oxygen species pathway
Interferon-α response
Interferon-γ response
Inflammatory responce
Unfolded protein response
**Biocarta pathways**
MHC pathway
IL-10 pathway
IL-3 pathway
RELA pathway
PCAF pathway
TNFR2 pathway
NTHI pathway

## Discussion

SS samples might represent a suitable totally non-invasive alternative to IS for the assessment of inflammatory biomarkers in the airways of COPD patients. In our study we set up and validated a processing-free method to stabilize SS samples at ambient temperature and isolate RNA with sufficient quality for downstream sequencing analysis. This methodology might allow the characterization of the inflammatory profile in the sputum of COPD patients also in conditions where specific know-how and capabilities for sample processing are missing, for example in large multi-center clinical trials.

This study originated from the findings that unprocessed SS (*SS_whole*) collected from COPD patients in an earlier randomized controlled trial (RTC) (Singh et al., 2019) did not show suitable quality for RNA sequencing analysis despite samples being immediately frozen at −80°C and transferred to the bioanalytical laboratory in dry ice. Following these observations, we investigated if stabilization of SS samples using a commercially available kit would preserve sputum sample quality for eventual gene expression analysis. To this purpose we used PrimeStore® MTM a proprietary blend of reagents that has been shown to efficiently lyse biological pathogens, stabilize and protect lysed RNA polymers from hydrolysis, oxidative damage or nuclease degradation and preserve RNA at ambient temperature for prolonged periods (Daum et al., 2011). We also evaluated if isolation of sputum plugs from saliva and other residues would significantly improve the transcriptomic outcomes.

SS samples were collected from COPD patients, stabilized in MTM with or without selection of plugs (*SS_MTM_Plugs and SS_MTM_whole*, respectively) and kept stored for 8 days at room temperature to mimic clinical settings in which sputum is transferred from sites to the laboratory at ambient conditions. In this context the RNA obtained from *SS* samples showed a quality similar to the RNA derived from FFPE samples with RNA integrity number (RIN) reduced to a level considered an indicator of degraded RNA (Wimmer et al., 2018). RIN values from degraded samples are not a sensitive measure of RNA quality nor are they a reliable predictor of successful library preparation. In these conditions the best indicator of RNA integrity assessment is considered the DV_200_ metric which accounts for the percentage of RNA fragments higher than 200 nucleotides (Matsubara et al., 2020). DV_200_ of at least 30% is generally recommended for downstream sequencing analyses (Illumina). Differently from samples collected without the use of stabilizer (*SS_whole*), RNA extracted from *SS_MTM_Plugs* and *SS_MTM_whole* samples showed an average DV_200_ higher than this threshold. In terms of quantity, 70% of these samples had an RNA yield >50 ng which is the recommended minimum input for progression to the total RNA sequencing method applied. Notably, in our experimental settings RNA was extracted from approximately 1.2 ml of the sputum and PrimeStore® MTM sample, suggesting that a higher initial loading volume would further increase the number of samples suitable for library preparation and sequencing.

Following RNAseq analysis we showed a straightforward correlation in terms of gene expression between *SS_MTM_plugs* and *SS_MTM_whole* samples. These findings were also confirmed by PCA analysis and supported the use of either method, with or without selection of plugs, to obtain adequate samples for RNAseq analysis of SS. PCA analysis highlighted only one sample pair with low matching metrics. Of note, both samples were associated to a lower mapping rates and quality in comparison to the other sample pairs. This once again emphasize the importance of DV_200_ quality metric to identify suitable samples for progression to sequencing. Overall, the correlation observed in this study between samples with or without selection of plugs has important operational implications since it indicates that selection of plugs which is a procedure requiring adequate know how and technical skills does not improve the transcriptomic outcome.

To assess whether the expression profile obtained for SS stabilized in MTM accurately reflected the typical inflammation of COPD patients in the airways, we characterized the enriched biological entities in our samples by ssGSEA. Further, we validated these findings by using processed IS samples collected from patients of similar clinical characteristics which were known to provide an excellent interpretation of the inflammatory expression conditions (Govoni et al., 2020). ssGSEA facilitates the interpretation of the biological profile of each sample transforming a single sample’s gene expression profile into a gene set enrichment profile. The enrichment score for each gene set represents the activity level of that particular biological process in which the gene set’s members are coordinately up- or down-regulated. ssGSEA results showed that 80% of the top 10 enriched pathways from *SS_MTM_plugs* and *SS_MTM_whole* samples were in common, confirming the significant high correlation between the two handling methods, with or without separation of plugs. For these samples key pathways associated to features of COPD inflammation, for example interleukins and interferon response, had top enrichment scores. Moreover, the top 10 enriched pathways resulting from ssGSEA of SS stabilized in MTM and IS samples showed a straightforward overlap with up to 70% of biological pathways in common. All of these pathways were associated to features of COPD, namely, systemic inflammation, immune responses and oxidative stress (Holguin, 2013; Rovina et al., 2013; Lopez-Campos et al., 2016). In particular, the ssGSEA results highlighted common pathways in terms of cytokine signaling (IL-6 and IL-10) (Silva et al., 2018), inflammatory signaling such as the nuclear factor kappa B (NFκB) activation through tumor necrosis factor alpha (TNF-α) receptors (TNFR) (Rovina et al., 2013), immune response signaling (T cell activation, MHC pathway and Interferon α and β) and reactive oxygen signaling (Holguin, 2013). This analysis confirmed that the expected endotype, typical of COPD patients is represented within the SS samples and indicates that stabilization of SS in MTM might be a suitable method to assess the level of sputum cell inflammation in COPD patients by RNAseq analysis.

While the validation of ssGSEA using IS samples from an independent cohort of patients represent a strength of the present investigation, the study has also important limitations. First, we collected IS and SS samples from different patient populations although with similar clinical characteristics. Second, the number of SS samples investigated was limited and based on this, we selected the average DV_200_ values within each collection method instead of the individual DV_200_ values as quality metric for progression to sequencing. Despite most of the samples having individual DV_200_ values > 30%, one sample pair showed DV_200_ consistently below this limit; yet, inclusion of this sample pair did not change the overall output of the ssGSEA analysis. Third, SS was not processed for cell counts or microbiome analysis and information on cell and microbial composition as possible quality measures were not available. However, the transcriptomic profile generated from SS samples was validated against that of IS samples of high-quality metrics. These samples were collected and processed in a RCT under highly standardized protocol conditions leading to an average viability of sputum cells of 92.5% and with high RNA quality (Govoni et al., 2020).To the best of our knowledge this is the first study showing suitability of *SS* samples in COPD patients to derive appropriate RNA for gene expression analysis. The implication of the current findings is particularly relevant in the context of large multi-centre studies, in which specific know-how and capabilities for sample processing are generally missing. Collection of SS with no further processing requirements eases the burden for patients and facilitates technicalities at sites. In addition, the possibility to transfer samples to bioanalytical laboratories at ambient temperature is dramatically reduces the high logistics costs typically associated to shipments in dry ice. These conditions might allow the evaluation of the pharmacodynamic profile of drugs in relation to clinical efficacy or the characterization of different endotypes for precision medicine approaches in large long-term clinical trials. Prospective implementation of these assessments in clinical studies is deemed necessary to validate further the methodology.

## Data Availability

Chiesi commits to sharing with qualified scientific and medical Researchers, conducting legitimate research, patient-level data, study-level data, the clinical protocol and the full clinical study report of Chiesi Farmaceutici S.p.A.-sponsored interventional clinical trials in patients for medicines and indications approved by the European Medicines Agency and/or the US Food and Drug Administration after 1st January 2015, following the approval of any received research proposal and the signature of a Data Sharing Agreement. Chiesi provides access to clinical trial information consistently with the principle of safeguarding commercially confidential information and patient privacy. To date, the current study is out of scope of the Chiesi policy on Clinical Data Sharing. Other information on Chiesi’s data sharing commitment, access and research request’s approval process are available in the Clinical Trial Transparency section of http://www.chiesi.com/en/research-and-development/. The data presented in the study are deposited in the GEO repository, accession number GSE175829 and GSE133513. The data is released and publicly available.
